# A Review of Automated Microinjection of Zebrafish Embryos

**DOI:** 10.3390/mi10010007

**Published:** 2018-12-24

**Authors:** Yuliang Zhao, Hui Sun, Xiaopeng Sha, Lijia Gu, Zhikun Zhan, Wen J. Li

**Affiliations:** 1 School of Control Engineering, Northeastern University, Qinhuangdao 066004, China; zhaoyuliang@neuq.edu.cn (Y.Z.); gulijia@stumail.neu.edu.cn (L.G.); 2 School of Electrical Engineering, Yanshan University, Qinhuangdao 066004, China; zkzhan@ysu.edu.cn; 3 Department of Mechanical Engineering, City University of Hong Kong, Kowloon, Hong Kong 999077, China; 4 Shenzhen Academy of Robotics, Shenzhen 518000, China

**Keywords:** Zebrafish embryo, cell microinjection, automated microinjection, microscopic visual servoing

## Abstract

Cell microinjection is a technique of precise delivery of substances into cells and is widely used for studying cell transfection, signaling pathways, and organelle functions. Microinjection of the embryos of zebrafish, the third most important animal model, has become a very useful technique in bioscience. However, factors such as the small cell size, high cell deformation tendency, and transparent zebrafish embryo membrane make the microinjection process difficult. Furthermore, this process has strict, specific requirements, such as chorion softening, avoiding contacting the first polar body, and high-precision detection. Therefore, highly accurate control and detection platforms are critical for achieving the automated microinjection of zebrafish embryos. This article reviews the latest technologies and methods used in the automated microinjection of zebrafish embryos and provides a detailed description of the current developments and applications of robotic microinjection systems. The review covers key areas related to automated embryo injection, including cell searching and location, cell position and posture adjustment, microscopic visual servoing control, sensors, actuators, puncturing mechanisms, and microinjection.

## 1. Introduction

### 1.1. Background of Automated Microinjection 

**Figure 1:**
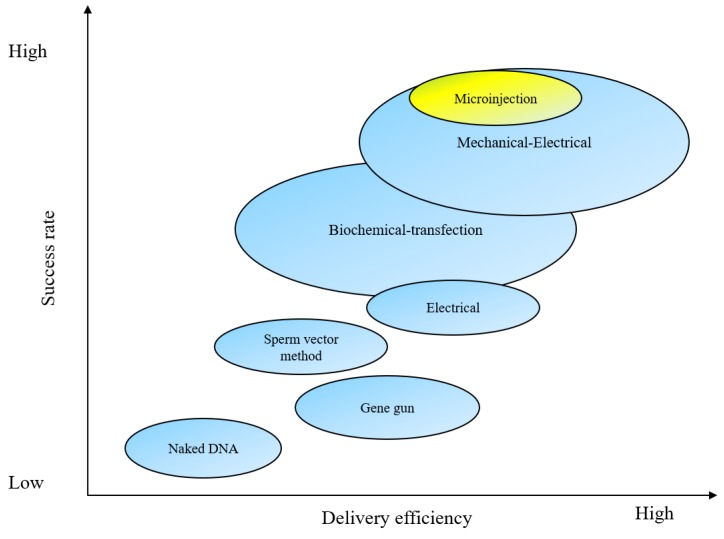
A comparison of the efficiency and success rate of delivering exogenous substances into cells by different methods (Courtesy of [15,16,17,18,19,20]).

Microinjection [1,2] is a technique of introducing exogenous substances, such as DNAs, RNAi, sperms, proteins, and drug compounds, into cells using a fine-tipped needle. It has been widely used for studying different aspects of living cells, from signal transduction to cell genetic structure and gene expression. Compared with other traditional physical delivery methods, such as electroporation [3,4], viral vectors [5,6], gene guns [7], ultrasound-mediated delivery [8,9], sperm-mediated delivery [10], hydrodynamic delivery [11,12], and dielectrophoresis (DEP) [13,14], microinjection is more toxicity-sparing and can maintain the biological activity of cells. It can be used for various target cell types, such as *Drosophila* (fruit flies) embryos, mouse embryos, and zebrafish embryos. The efficiency and success rate of delivering exogenous substances by the traditional methods are shown in Figure 1.

Zebrafish is one of the most well-established research models in life sciences and biotechnology. They are relatively transparent at the embryonic stage, which facilitates the observation of early morphological changes. They are ideal for DNA or mRNA injection, cell labeling, and transplantation. Therefore, zebrafish embryo microinjection (ZEM) has been widely used in many fields, such as genetics [21], virology [22], toxicology [23], endocrinology [24], immunology [25], and oncology [26]. It is playing an essential role in advancing the field of cell biology, such as in genetics, transgenics, assisted reproduction, and drug discovery. However, conventional microinjection techniques are time consuming and error prone and have a low success rate. With the development of microscopic vision, micro-nano manipulation, mechanical engineering, and servoing control, automated ZEM has been realized as an alternative to manual or semi-automated methods.

**Figure 2:**
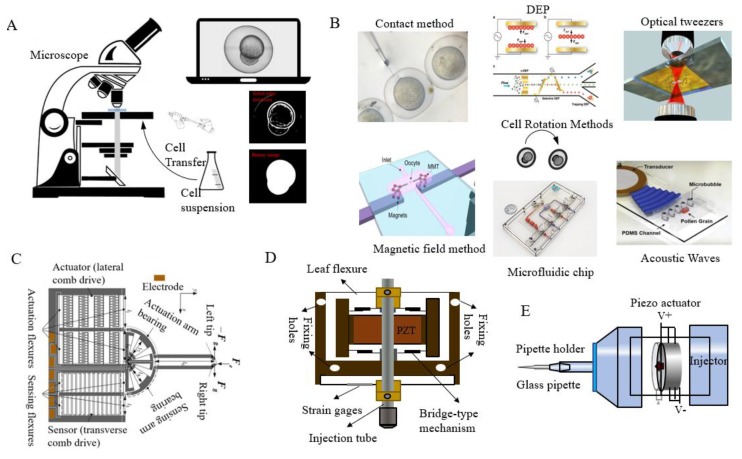
Key parts of a microinjection system: (**A**) cell manipulation and detection methods, (**B**) cell posture adjustment, (**C**) sensor detection (Courtesy of [34]), (**D**) needle actuator (Courtesy of [35]), and (**E**) injector (adapted from [36]). DEP: dielectrophoresis.

Figure 2 shows the main parts of an automated microinjection system. In recent years, several research groups [27,28,29,30,31] have attempted to develop automated ZEM using technologies such as computer processing, microscopic image processing, servoing control, and micromachining. However, most injection strategies still rely on a holding pipette to immobilize a single cell, which greatly limits the efficiency of the cell injection process. Some automated suspended cell injection strategies [30,32,33] are complicated to use and involve a time-consuming injection process.

### 1.2. Key Issues in ZEM

In addition to the abovementioned issues with detection control methods, techniques and the characteristics of zebrafish embryos membrane in development should also be considered. The chorion softening process that occurs during the early development of zebrafish embryos [37,38] changes the quantitative relationship between the applied force and the deformation of the chorionic structure at different embryonic stages. This greatly affects the puncturing mechanism of the microinjection needle. Furthermore, there are strict requirements regarding the adjustment of the cell position during the zebrafish embryo injection process, i.e., the microneedle should not contact the first polar body during injection. The animal pole, i.e., the pole with less yolk and faster cleavage, is the ideal site for embryo injection. 

Therefore, based on the abovementioned characteristics and operational requirements of zebrafish embryos, the following developments in the automated ZEM process are warranted: (1) to avoid structural damage to the cells and effectively improve the efficiency of the operations, a system and method capable of immobilizing a large number of zebrafish embryos and rapidly detecting their position must be developed; (2) an automated and robust system for detecting and adjusting the cell posture based on visual servoing control must be developed, which will help avoid cell posture adjustment during the pre-piercing stage; and (3) the two driving devices must be coordinated to quickly and effectively perform cell puncture and quantitative injection, so as to ensure that the changes in the relationship between the applied force and the deformation of the chorionic structure caused by chorion softening during zebrafish embryo development do not affect the puncturing mechanism.

### 1.3. Current State of Experimental Research on ZEM

The problems encountered in the various steps of microinjection and their solutions are described in Table 1. Several solutions to the listed problems have been reported by many research groups. To immobilize a large number of embryos, X. Liu [39] established a multi-micromanipulator system to rapidly detect the embryos and obtain three-dimensional (3D) positions of the cells and manipulators. For visual servoing control and cell posture adjustment in automated microinjection, W. Wang et al. [40] proposed a robotic micromanipulation system based on computer vision and motion control. Z. Nan et al. [41] developed a robotic microinjection system for cell recognition and path planning based on a pattern matching method and genetic algorithm. Z. Wang et al. [42] designed a fully automated 3D cell-rotating robotic micromanipulation system that can rotate a single zebrafish embryo to the desired orientation by fluidic flow control using motion control and computer vision. To address the problems in cell injection and puncturing, H. B. Huang [36] designed a piezo-driven cell injector, and Z. Lu et al. [43] designed a prototype system for batch injection of zebrafish embryos. This system can guide the micropipette to penetrate the embryo at a rapid but constant rate.

**Figure 3:**
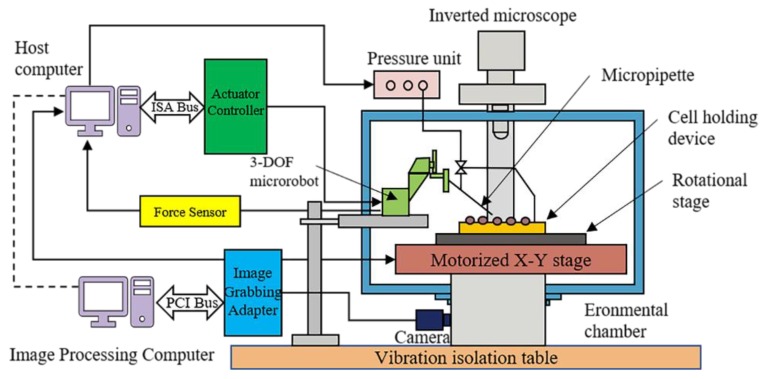
Schematic of a typical automated microinjection system (adapted from [88]).

The following sections provide an analysis of recent studies on these topics and a summary of the advantages and limitations of their proposed solutions. Covered in the following sections are also some suggestions for improving the automated ZEM. A typical automated microinjection system is shown in Figure 3. 

## 2. Cell Searching and Positioning

The first step of microinjection involves searching for the cells in a microscopic field and proper positioning of the yolk centroid using the motion servoing control system to achieve the target position for puncturing with the injection needle. Image processing is the most important technique involved in searching and positioning, especially for cell and needle identification and autofocusing. It can be divided into image preprocessing, image segmentation, object recognition, and image understanding stages, the most critical stages among which are image segmentation and object recognition [41]. In the following sections, we describe the applications of these image processing techniques in cell searching and positioning.

### 2.1. Cell and Injection Needle Identification

**Figure 4:**
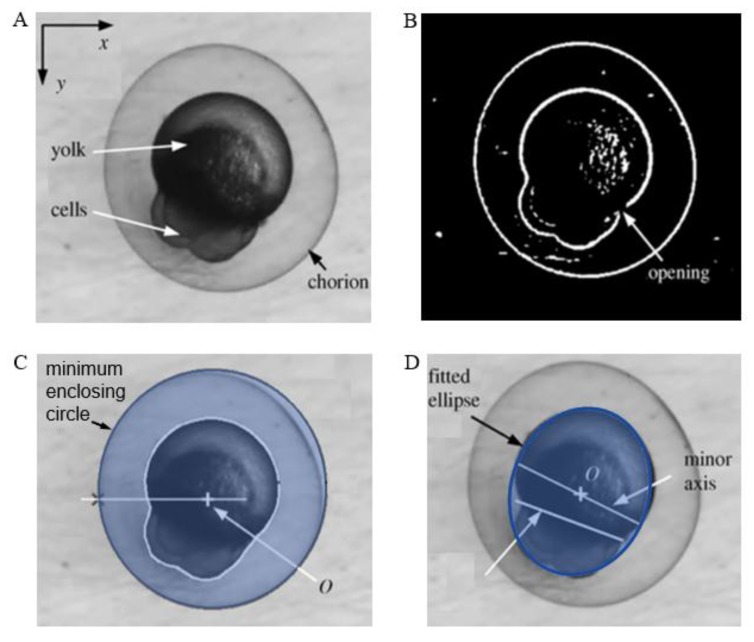
Identification of zebrafish embryo structures: (**A**) zebrafish embryo; (**B**) zebrafish embryo after preprocessing; (**C**) identified chorion, cytoplasmic center, and switching point; and (**D**) distinguished yolk and cell portion (Courtesy of [97]).

In the cell recognition process, image segmentation is used to divide the cell image information into foreground and background, which helps to derive valid information about the contours and edges of the cell. To date, many comprehensive studies have been conducted on image segmentation [89]. The existing segmentation algorithms can be roughly classified into three types. The first type comprises the image-based edge detection methods, including the Canny algorithm [90], Hough transform [91], Sobel algorithm, and snake model [92]. The second type comprises methods based on the statistical characteristics of gray histograms, including the adaptive threshold segmentation algorithm [93,94], Ostu algorithm [95], and fuzzy threshold segmentation. The third type comprises methods based on region-based segmentation algorithms, including the split merge algorithm and the regional growth algorithm. Because zebrafish embryos are generally considered to be in a spherical or approximately spherical shape, they are often identified by the Hough transform [47,50,51,52,96]. This method enables faster calculation and more efficient recognition than other methods. However, the problem with this method is that during the binarization process, the target object may be mixed with certain areas of the environment and thus display several erroneous edges in the image. In addition, cells are inevitably stacked together in the cell recognition process, so it may become difficult to explain their circular characteristics once the image is converted into the binary form. Figure 4 shows the identification process of zebrafish embryos.

Real-time tracking of the needle is also a key part of the microinjection process. There are three types of microscopic vision-based tracking methods: image template matching-based tracking, feature point-based tracking, and active contour-based tracking. The image template matching-based method usually delivers high tracking accuracy but does not work well when the target is occluded, the target shape and size change, or any rotation occurs. The active contour-based tracking method (i.e., Snake tracking [98]) involves a typical image tracking model. In this method, the given initial contour gradually approaches the actual target contour under virtual force, and real-time tracking of the target is achieved by making the shape and position of the target contour change dynamically.

Due to its fixed shape, the injection needle does not deform, nor does it disappear completely when blocked. Therefore, in microinjection needle recognition algorithms, the cross-correlation template matching [50,96] method is usually adopted to identify the micro-glass needle for cell injection. The template matching method divides the needle tip region from the original image to function as a needle tip template [48,55]. This template is then matched against the whole image to detect the target with a satisfying level of error. However, the gray image-based template matching method tends to be time consuming.

### 2.2. Autofocusing

An autofocus system can rapidly obtain high-quality image information, which lays a solid foundation for subsequent cell microinjection processes, such as image processing and precise positioning. Autofocus is an essential step in achieving automated microscopic visual micromanipulation. Autofocus technology covers three aspects: image sharpness evaluation function, focus position search, and image sharpness global maximization search strategy.

G.H. Zong et al. [99] proposed two focusing functions related to the wavelet transform that are based on discrete wavelet transform- and continuous wavelet transform-based autocorrelation. B.J. Yu et al. [100] used the target region selection method to combine the two sharpness evaluation methods to achieve a full closed-loop feedback of the positioning system. L.G. Chen et al. [101] adopted a focusing method based on depth from defocus. Z.Q. Zhou et al. [102] proposed an autofocus and control method that uses a combination of the lifting wavelet transform and the Sobel edge detection operator to form a focus evaluation function and the self-organizing algorithm to perform unsupervised training on the focused and defocused images. 

Although the image processing-based autofocus technology is becoming increasingly popular in optical imaging systems, some improvements are required in the existing methods:

- There are local extreme points. The actual focusing process is prone to falling into the local extremum and thus causes the focusing to fail. This problem is usually solved by improving the focus search strategy and finding an ideal focus evaluation function.
- Objective quantitative evaluation metrics are unavailable for evaluating the focusing function, and the specific value cannot be reflected by the performance of the focus curve function.
- It is difficult to balance speed and accuracy in the focus search strategy. The small size of the target object requires high accuracy, which is difficult to meet while operating at the required speed.

### 2.3. Cell Posture Adjustment Methods

To enable injections at specific locations of the embryo and to avoid any damage to specific internal organelles, the position and posture of the cells are often adjusted using mechanics, fluid electric fields, and magnetic fields.

Cell position and posture adjustment usually involves using a holding pipette to aspirate the zebrafish embryos and keeping the injection needle away from the first polar body during the injection process. The cell’s animal pole is the ideal injection site, but it is also possible to target the yolk for direct injection and let the sample be distributed into the cells with the flow of the cytoplasm and the yolk. A fast, reliable, and precise method for cell position and posture adjustment can be a stable aid to subsequent operations, such as membrane puncture with a microinjection needle. To facilitate this, many micro-nano robot control systems and physical methods have been proposed to adjust the cell posture. Currently, the cell position adjustment methods can be roughly divided into two categories: contact and non-contact methods. A summary of these methods is provided in Table 2.

#### 2.3.1. The Contact Method

A mechanical contact method is the most direct and effective method of performing cell position adjustment using micro-robots or micro-manipulation tools [103,104]. Given the particularity of zebrafish embryos and the experimental environment, the micromanipulator must meet several strict requirements. Zebrafish embryos are structurally weak, so a reasonable amount of force should be applied to avoid damaging the embryos. The micromanipulator must be adapted to the operating environment to overcome the scale effect of micromanipulation. Therefore, the micromanipulator should be able perform fine operations within a limited space and in a manner that keeps the target object in the microscopic field. Moreover, the micromanipulator should have multiple degrees of freedom, operational flexibility, ease of adjustment, and high motion accuracy to ensure precise cell position and posture adjustment. To increase the accuracy and repeatability of the system, force feedback and visual feedback can be introduced. One example of a mechanical contact method is an automated cell rotation system proposed by Z. Wang [60] that uses a three-point contact method to manipulate a single cell.

#### 2.3.2. The Non-Contact Method

The non-contact method, which has also become popular among researchers, can be performed by two mechanisms. One is by creating a microfluidic flow for the solution in which the cell is guided to move or rotate (e.g., the microfluidic method [42,105,106,107,108,109,110]). For the distinctive non-contact method to work properly, a closed space is required to ensure minimal interference from the external environment. The other is by applying a type of “force” or “torque” directly onto the cell and allowing it to move or rotate (e.g., DEP [111,112,113,114,115,116,117,118,119,120,121,122,123,124], the electromagnetic method [125,126,127,128,129], and the acoustic wave method [130,131]).

### 2.4. The Microfluidic Method

The microfluidic method based on the flow characteristics of fluids controls the cell position and posture by generating a controllable microfluidic field at microscale. This method guides the cell movement by the pressure and viscosity generated by the fluid motion. The disadvantage of this method is that changes in the cell size and the varying viscosity of the solution can affect the cell rotation result.

**Figure 5:**
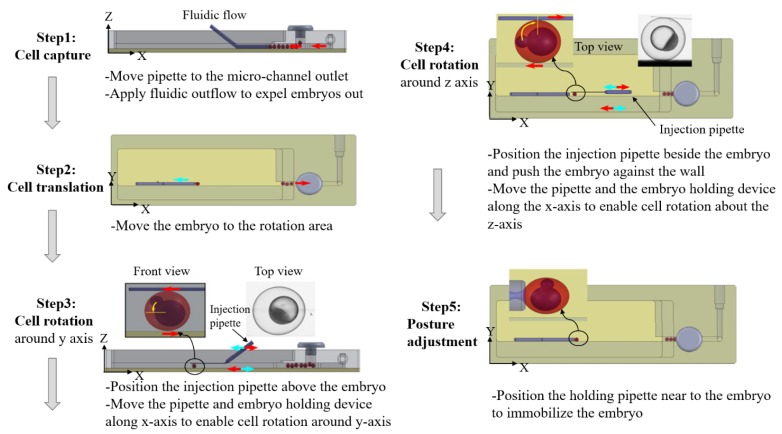
Sequence of the steps involved in of the microfluid cell orientation method (Courtesy of [107]).

N. Chiba et al. [105] designed a small-scale system that works in a novel way to enable cell position and posture adjustment. Although this method does not cause any mechanical damage to the cell, the inability of the flow field generated by the oscillations of the glass microrods to control small-sized cells makes it less applicable. In addition, the experimental control parameters, such as the distance between the glass microrod and the cell as well as the oscillation frequency and amplitude, often need to be redetermined as the cell size changes, which is cumbersome and difficult. Z. Wang [107] used a pair of standard micropipettes and an internally fabricated embryo holder to avoid the above problems. A schematic diagram of this experimental process is illustrated in Figure 5.

In summary, the microfluidic method does not damage the cell, but its experimental efficiency is low due to a complicated debugging process and an uncontrollable operation process.

### 2.5. DEP

**Figure 6:**
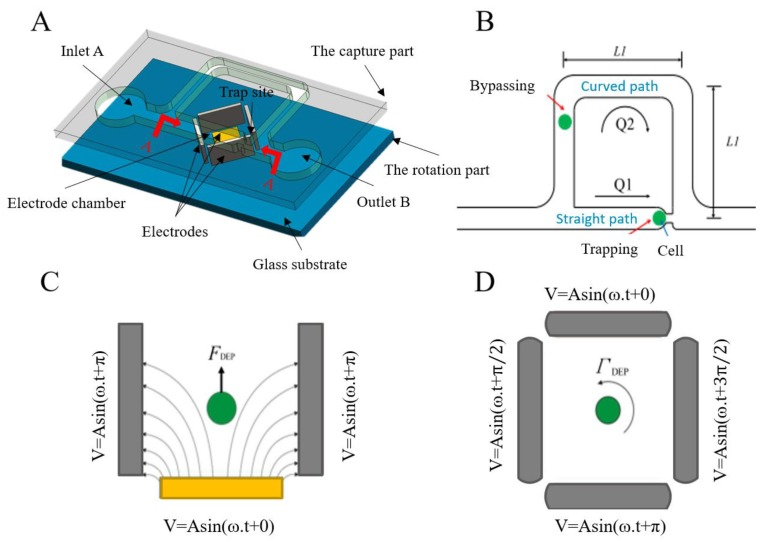
The design and operational principles of an integrated dielectrophoresis (DEP) chip: (**A**) a decomposition diagram of the comprehensive model; (**B**) a schematic diagram of microchannels; (**C**) the top view of the electrode chamber on the DEP torque, and (**D**) the lateral view of the electrode chamber with respect to the DEP forces (Courtesy of [111]).

DEP is a preferable cell rotation method due to its advantages of fast manipulation and high-precision position adjustment. In DEP, non-conductive objects are moved to different degrees by polarization under an external electric field. When the cell is in a non-uniform field strength and is thus under the influence of varying electric field forces, its position and posture can be adjusted using two pairs of electrodes. A typical DEP cell rotation method is shown in Figure 6.

F. Arai et al. [117] developed a cell micromanipulation system that uses a rotating electric field driving technology to simultaneously adjust the position and posture of a group of cells. J. Park et al. [114] used microfluidic channels prepared by photolithography to transport cells, in which the cells are rotated using a rotating electric field technique. C.P. Jen et al. [115] designed a microchip with an open-top microstructure for insulator-based DEP capture. T.P. Hunt [116] proposed the use of DEP tweezers to capture cells. C.C. Wang [118] designed a micro-scale particle trap with an improved planar structure integrated in a biochip system. L. Huang [123] proposed a novel, efficient chip for single-cell loading and 3D cell rotation.

The disadvantage of DEP in adjusting the cell position and posture is that the effect of the electric fields on the cells is unclear, and it is often difficult to construct and debug a system in which the cells are rotated in two orthogonal planes.

### 2.6. The Magnetic Field Method

**Figure 7:**
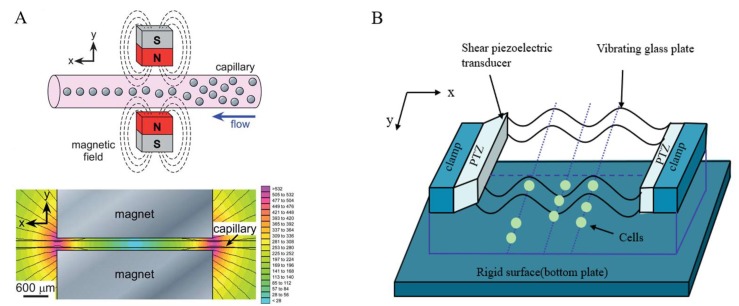
(**A**) Cell position and posture adjustment by rotation using a magnetic field. (Courtesy of [129]); (**B**) a schematic diagram of ultrasonic cell manipulation (Courtesy of [132]).

The magnetic field method is based on the principle that magnetic dipoles are arranged in a disorderly manner when they are not affected by any external magnetic field. When a magnetic field is added around the material, the magnetic dipoles point in the same direction and exhibit magnetic properties. Therefore, the position and posture of the cells can be adjusted by changing the force acting on them in the magnetic field. A. Winkleman et al. [127] verified this principle using three pairs of electromagnetic poles to generate a rotating magnetic field around the cells; the magnetic force could rotate the cells to the desired position and posture. In addition, S. Floyd et al. [128] proposed a method that uses an external magnetic field to control a tiny magnetic tool to indirectly change the cell position and posture. Figure 7A shows the use of a magnetic field to rotate the cell to the desired position and posture.

Similar to DEP, the disadvantage of the magnetic field method is that the effect of the magnetic field on the cells is unknown, and it is difficult to construct and debug the system.

### 2.7. The Ultrasound Method

The ultrasound method for cell position and posture adjustment works by controlling one or more tiny objects suspended in a culture solution without prior positioning. This method is typically used to capture tiny objects based on the superposition of pressure waves in two orthogonal planes. N. Läubli [131] used an acoustic wave-based microfluidic device that generates a local vortex by resonant acoustic excitation of air-filled microbubbles, allowing the cells to rotate in a controlled 3D space. However, when the ultrasonic wave in the liquid reaches a certain intensity, cavitation occurs, which consequently produces a high local temperature and pressure. This method is inefficient, especially in controlling cells at high-density. Therefore, further experimental research is required to verify the applicability of this method in the biological field. The improved acoustic method controls the cell position as shown in Figure 7B. D.H. Kim [132] proposed a novel high-throughput cell manipulation method using acoustic wave technology; this method can effectively perform cell capture and transfer. 

## 3. Microscopic Visual Servoing System

The task of microscopic visual servoing is designed for controlling the position of the micromanipulator’s end-effector by automatically acquiring and analyzing the information from the image to form a closed loop. In the automated ZEM, the needle should move toward the target location from where the cell is to be extracted, so as to facilitate the subsequent cell injection task. The system should enable accurate and rapid movement of the injection needle to the target position without positional overshoot that would cause the needle to touch other cells or damage the cell being injected, thereby hampering the success of the experiment. The proportional-integral-derivative (PID) controller, one of the most commonly used control system in engineering applications, is also suitable for micromanipulation [133]. In this system, the upper computer guides the mechanical arm to move the microinjection needle to the target position based on the PID control law and releases the injectable substance. This is a very coarse-grained control strategy because it requires only steady-state performance, i.e., reaching the specified position, but ignores the dynamic effects caused by target deformation during the needle movement and injection.

**Figure 8:**
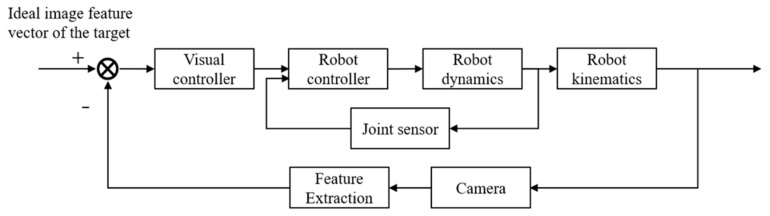
Diagram of an image-based visual servoing control structure.

Considering the particularity of the cell injection environment, an image-based dynamic look-and-move visual servoing control structure can be a suitable choice for robotic manipulation systems [97,134]. For optical microscope-based vision systems, the camera can only be mounted on the microscope for simultaneously observing the target object and the end-effector. Therefore, good injection results can be obtained using an end-point closed-loop system as shown in Figure 8.

The advantages, disadvantages, and problems of image-based visual servoing control are listed below:

- Because a closed loop is formed in the image space, image-based visual servoing control is insensitive to calibration and spatial model errors to ensure a high level of control precision. The error is calculated directly in a 2D image space, without requiring any 3D reconstruction.
- Image-based visual servoing control is generally required to calculate the depth information of the target, to calibrate the internal and external parameters of the camera, and to perform hand–eye calibration. In particular, the image Jacobian matrix is difficult to obtain, and the inaccuracy of this matrix also makes it difficult to perform stability analysis on the system, which hinders the controller design process and leads to control system instability.
- The selection of image features has a significant effect on how well the control system performs. Selecting image features with high robustness and weak coupling is a key consideration in working with an image-based visual control system.

In addition to the commonly used image-based visual servoing approach, force-based [135], position-based, and position- and force-based hybrid servoing control [44,136] approaches are well suited for controlling the motion of microinjection needles. These approaches can effectively prevent the cells from being damaged by the excessive injection force of the microinjection needle and positional overshoot. Experiments have shown that compared with the traditional control techniques, the hybrid servoing control system has a faster response time, higher precision level, and higher success rate.

## 4. Actuator

An actuator enables the micro-displacement driving mechanism to move within an appropriate range during the cell microinjection process and prevents the cell from being damaged. The actuator is mainly used as a microinjection manipulator to enable precise and rapid movement of the microinjection needle in and out of the cell, and the length of time the capillary tip remains in the cell directly affects the intracellular injection volume. The zebrafish embryo to be manipulated generally has a diameter of approximately 1 mm. This small size warrants a high-precision microinjection system. Therefore, it is crucial to use a suitable actuator as the needle manipulator. Depending on the type of driving force, actuators can be roughly divided into the following categories: piezoelectric type, pneumatic type, shape memory effect, electrostatic force, linear motor, mechanical drive, electrothermal, electromagnetic force, and laser drive. Table 3 summarizes the advantages and disadvantages of some of these driving mechanisms. The stacked piezoelectric ceramic actuator, a new type of micro-displacement device, is considered as an ideal choice for cell manipulation due to its advantages of large driving force, large displacement, high rigidity, and fast response. It is particularly applicable for manipulating zebrafish embryos because of the tough double-layer structure of their cell membrane and zona pellucida, which require an actuator that minimizes the damage to the cell to ensure a high success rate of injection. However, the actual output of the piezoelectric ceramic actuator is not ideal because the output displacement is not linear with the input signal in the strict sense, and the actuator exhibits problems such as creep, hysteresis, and nonlinearity.

## 5. Microsensor Detection System

Micro-displacement sensors, micro-force sensors, and vision sensors are commonly used in microscopic cell injection systems to obtain information on zebrafish embryos. Subsequently, the manipulation task is completed by extracting the best active embryos and guiding the injection needle to the optimal cell position by visual servoing control. The cell is very sensitive to the action of the microinjection needle and the holding pipette, so it is critical to accurately control the amount of force applied to the cell to prevent any damage. The microscopic system should contain a feedback system to obtain information on force or position. The common types of feedback include position feedback, visual feedback, force feedback, and tactile feedback. Despite offering visual micromanipulation and a greatly improved level of automation, the microscopic vision system may be occluded, rendering it unfit for determining the position and posture of the target. Thus, it is preferable to control the micromanipulation arm using displacement or force sensor-based feedback than using visual feedback. Currently, micro-force sensors are the most widely used sensors in microinjection systems for automated injection. They generate feedback parameters to measure the force applied by the manipulators and the injectors. The mechanical data collected directly during the injection process provide information on the physical characteristics of the injected cells, making the system easier to manipulate.

**Figure 9:**
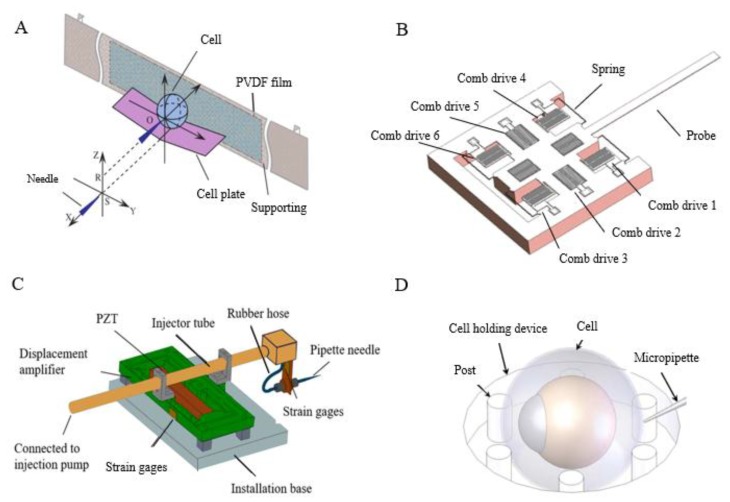
Several typical micro-force sensors: (**A**) beam structure of the polyvinylidene fluoride force sensor (type O contact position) (Courtesy of [158]), (**B**) solid model of the multi-axis cellular force sensor [66], (**C**) the strain-gauge position and force sensors [159], and (**D**) schematic configuration of vision-based cellular force measurement (Courtesy of [68]).

The current micro-force sensors can be categorized into six types: piezoelectric sensors, piezoresistive sensors, capacitive sensors, strain gauges, magnetic effect-based sensors, and vision-based sensors. The detection principles, advantages, disadvantages, and control accuracies of the various types of sensors are described in Table 4. Figure 9 shows several typical micro-force sensors. 

Two force measurement sensors, namely, capacitive and visual sensors, are commonly used in the microinjection systems. Micro-force sensors usually feature a cantilever beam structure made of a silicon wafer and a semiconductor detection circuit created at an appropriate position of the silicon beam by photolithography and etching in a microelectromechanical system (MEMS). K.P. Roberts [66] fabricated a two-axis capacitive micro-force sensor using a deep ion etching process. Y. Sun et al. [67] developed a MEMS capacitive force sensor that can not only measure a wide range of forces from mN to pN but also provides information on force along multiple axes. It has the advantages of low power consumption, low noise, and high sensitivity. X. Liu et al. [68,69] invented a device that uses six low-stiffness elastic posts to fix cells. It can indirectly obtain information on the force applied to the cell using the microscopic vision method. D.H. Kim [38,70] developed a piezoelectric cell pressure sensor that uses a polyvinylidene fluoride (PVDF) film as a pressure-sensitive component and the inverse piezoelectric effect of the PVDF film for measurements. The PVDF film pressure sensor allows the measurement of the mechanical properties of zebrafish embryos at different developmental stages. S. Muntwyler et al. [72] developed a MEMS-based bulk silicon microfabrication process to build a three-axis force sensor with sub-micro-Newton measurement uncertainty and a tunable force range.

Existing micro-displacement sensors, such as micro-fabricated capacitive sensors, inductive displacement sensors, resistance strain displacement sensors, fiber sensors, grating sensors, and photoelectric encoders, generally use a MEMS-based technology to measure position or distance. F. Yang and Y. Ying [73] at Tsinghua University designed a microbeam displacement measurement method that integrates double-grating interference and a charge-coupled device image measurement system. X.J. Zhanga et al. [71] proposed an optical encoder based on a transmission phase grating combined with an injector to indirectly measure the injection force applied to *Drosophila* embryos. A.F. Ergenc [74] proposed a novel method to detect the tip displacement of a 3D glass pipette using laser optics. F. Karimirad [75] at Monash University in Australia proposed a vision-based force measurement method using a neural network model. Karimirad further studied the various stages of cell deformation during the injection of spherical cells by tracking and characterizing the deformation in real time using a dimple angle.

## 6. Characterization of Cell Models

**Figure 10:**
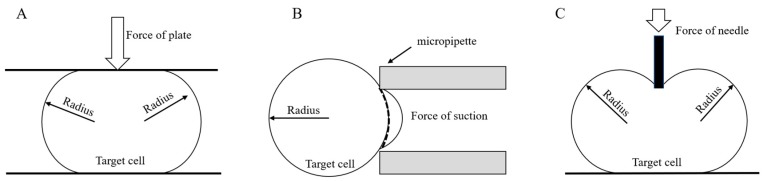
Diagrams of (**A**) compression between two plates (adapted from [160]), (**B**) micropipette aspiration (adapted from [161]), and (**C**) a point load model (adapted from [66]).

The change in the quantitative relationship between the applied force and the deformation of a chorionic structure caused by chorion softening during the embryonic stage is a major obstacle to successful injection. To overcome this obstacle, researchers often use a holding or squeezing method [160] in conjunction with various sensors to establish the relationship between the applied force and embryo deformation and determine the material and mechanical properties of the cell membrane. Figure 10 shows three typical methods for determining the mechanical properties of cell membranes. Y. Tan [76] proposed a mechanical model based on the membrane theory, which uses a quasi-static equilibrium equation to establish the relationship between force and biological cell deformation. Y. Sun et al. [37] used micro-sized micro-pillars obtained by micromachining to extrude embryos and measure the Young’s modulus of the embryonic membranes. Y. H. Tan [76] used a microinjection needle and a PVDF micro-force sensor to measure the Young’s modulus of the embryonic membranes and achieved ideal results. However, the biggest drawback of this extrusion-based membrane property measurement method is that it requires high-precision sensors capable of sensing micro-Newton-level forces, which are costly and require special customization. Therefore, it is critical to develop methods that can measure membrane properties based on visual feedback without relying on any special sensor.

A zebrafish embryo can be thought of as a liquid ball larger than a holding pipette and wrapped in a layer of membrane. The embryo fits well with the model for measuring the membrane shear modulus of cochlear outer hair cells based on the aspiration method described previously [77,78,161]. Therefore, this model can be used to measure the shear modulus of the zebrafish embryonic membranes. The relationship between the aspiration pressure and the length of the embryonic membrane aspirated by a holding pipette can be automatically obtained using an image processing algorithm. Furthermore, the relationship between the aspiration depth, the aspiration pressure, and the shear modulus of the zebrafish embryo can be obtained using this model. 

D. Sun et al. [32] and Y. Sun et al. [66] proposed a cell membrane point load model to determine the relationship between the applied force and cell deformation. H. Ladjal et al. [162] demonstrated a dynamic modeling method that uses finite element mechanics to simulate cell deformations in real time. This method provides an intuitive and reliable way to study the forces applied on cell membranes. All of the above methods provide a relationship between the cell membrane and the applied force, ensuring that the cell membrane is pierced with a suitable force and the success rate of injection is improved.

## 7. Puncture and Injection

**Figure 11:**
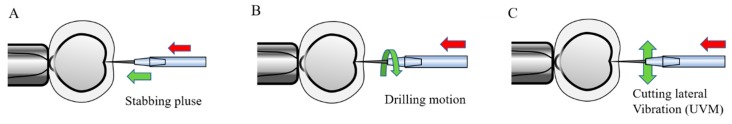
Puncturing approaches based on a piezo-driven pipette with (**A**) stabbing pulse movements (adapted from [81]), (**B**) drilling movement (adapted from [82]), and (**C**) lateral vibration movement (adapted from [83]).

To introduce foreign substances into a cell at a specified position, the microinjection needle must first puncture the outer transparent band or the cell membrane to penetrate the cell. This process requires the microinjection needle to move with a fast instantaneous speed and a high positioning accuracy to avoid excessive cell deformation or damage to the cell membrane. In particular, the deformation of the chorionic structure varies at different embryonic development stages of the zebrafish, and the elastic modulus of the chorion can be determined using the biofilm elastic model. The average force required to puncture the chorion at the blastula stage is 1.3 times greater than that at the prehatching stage. The elastic modulus of the chorion at the blastocyst stage is 1.66 [37] times greater than that at the prehatching stage. Considering these factors, the microinjection needle should satisfy the following requirements: (1) It should be capable of moving with a fast instantaneous speed to overcome the effect of cell membrane viscoelasticity, reduce cell deformation, and avoid excessive damage to the cell membrane. (2) It should have a positioning accuracy up to the submicron scale or even lower to ensure successful experiments. (3) A cell injection procedure involves rapid needle insertion, impact puncturing, precise positioning, injection, and withdrawal steps. Real-time control of the membrane puncturing process is crucial for enhancing the continuity and automation of each step. As shown in Figure 11, the puncturing mechanism is mainly implemented by three approaches: pulse-based puncturing, cutting vibration, and piezoelectric drill.

Y. Kimura and R. Yanagimachi [81] designed a pulse-based actuator for cell puncturing. Despite the high resolution and stability of the piezoelectric drive, the drive signal of the piezoelectric material produces an undesirable lateral motion at the tip of the needle, which can considerably damage the cells. To address this problem, H.B. Huang [36] designed an injection device that assembles a piezoelectric stack at the end of a piezoelectrically driven cell injector, which can reduce the vibration amplitude. In the ultrasonic cutting process of the test procedure [83], the cell sustains almost no deformation during cell injection. Compared with the traditional cell puncturing techniques, this method uses an ultrasonic cutting force instead of a penetration force to puncture the cell membrane, which provides better control over the movement speed during cell injection. The harmful lateral tip oscillations of the injector pipette can also be reduced. To address the oscillation problem, N. Olgac et al. [163] designed a cell puncturing device leveraging the rotational oscillations of the pipette tip. W. Johnson et al. [82] designed a flexure-guided piezoelectric drill for penetrating the zona pellucida of mammalian cells. The piezoelectric drill, operating under carefully selected and filtered pulse train signals, offers large axial and low lateral oscillation amplitudes and causes only a minor deformation during the penetration of the zona pellucida. 

Microinjection is considered to be the most effective method for delivering foreign substances into the cells due to its advantages of causing minimal damage to the embryos, ease of material preparation, and high injection efficiency. There are three basic microinjection techniques: capillary pressure microinjection [1], capillary electrophoresis [86], and capillary iontophoresis [87]. Of these, capillary pressure microinjection is the most commonly used technique in automated microinjection.

Capillary pressure microinjection [1] is a mechanical method that can be further divided into two subtypes: pulse-based pressure gas-driven microinjection [84,112] and equilibrium pressure microinjection based on the static principle method [45]. Many factors, such as the manipulation device, the cell itself, and the characteristics of the injection, may affect the microinjection process performed using this method. Therefore, some uncontrollable biological interference factors, such as intracellular pressure, membrane elasticity and size, injection viscosity, uniformity, and air bubbles, also affect the relationship between the injection parameters and the injection volume. 

In an injection model based on the pulse pressure method, the control of the injection volume from the microliter to the fly level can be realized. This method is fast and has high control precision but has two drawbacks: (i) The injection volume is difficult to be accurately controlled; and the quantitative injection repeatability is poor. (ii) In the injection process, the effect of the injection surface on the injection volume is not considered. Application of the same pressure for the same length of time on different injection surface positions is likely to cause a substantial deviation in the injection volume.

The equilibrium pressure injection method based on the static principle can accurately control the microinjection volume but is limited because its model is based on the premise that liquid is stationary. The equilibrium pressure model ignores the friction between the liquid and the tube wall, as well as other microscale forces that may cause changes in the state of the fluid motion. These simplified processes reduce the accuracy of the model.

**Figure 12:**
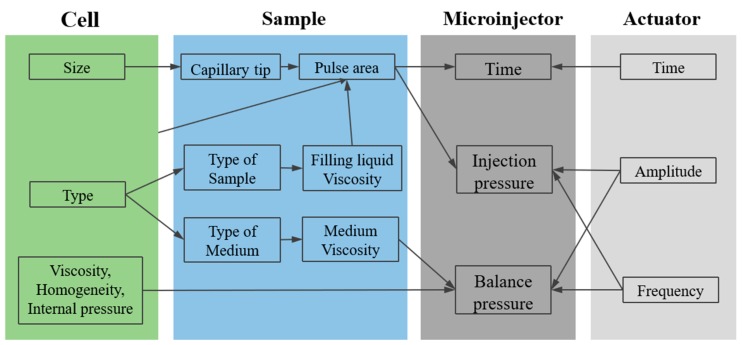
The relationship between injection parameters and interference (adapted from [1]).

The automatic quantitative injection method should be able to achieve automated quantitative injection control. Nanoscale quantitative injection is required for zebrafish embryos. When the injection operation is affected by the microscale effect, high flow resistance is likely to occur in the microinjection needle, thereby reducing the average flow rate of the injection (i.e., a low Reynolds number). Three key factors affect the injection volume, namely, the pressure difference applied to the front and rear ends of the injection surface, the opening radius of the microinjection needle, and the injection time. Therefore, it is necessary to establish an injection volume model considering these parameters [45,64,164,165] and to leverage intelligent parameter optimization to identify the model parameters. The factors that specifically affect the injection volume are shown in Figure 12. The injection volume can be controlled by the driving voltage amplitude, frequency, and driving times applied to the piezoelectric ceramics. However, there is one drawback to this model: As the parameters vary with substances of different viscosities and concentrations, the model needs to be re-established whenever any new substance is to be injected, which greatly reduces its general applicability. 

## 8. Conclusions and Prospects 

In conclusion, this paper comprehensively reviews recent studies on zebrafish embryo microinjection and showed that techniques for each step of the microinjection procedure have been fully developed for automatic ZEM. Nonetheless, the drawbacks of some techniques warrant further research to enhance the efficiency and success rate of injection. First, the immobilization and posture adjustment of cells in batches need to be accomplished to greatly improve the efficiency of microinjection. Second, position sensors with nanometer-level accuracy, force sensors that can detect force in the nanonewton range, and actuators that can handle picoliter-level injecting volumes are required to further reduce embryo damage during microinjection. Moreover, breakthroughs have been made in several relatively new interdisciplinary methods and techniques, such as microfluidics, MEMS sensors, and new control techniques, which could be combined to develop microinjection platforms with higher levels of precision and efficiency for automated microinjection operations. These technological advances will continue to provide researchers in biology, genetics, and medicine with access to automated microinjection approaches with high throughput, high efficiency and high success rates in the coming decades. 

## Figures and Tables

**Table 1:** Solutions to the problems encountered in the zebrafish embryo microinjection process.

Experimental Operations	Technical Parts Involved	Main Tasks	Key Problems to Resolve
1. Immobilize zebrafish embryos and detect their positions	Cell immobilization, cell detection, and tracking	• Cell immobilization [40,43,44,45,46] • Cell detection [41,47,48,49,50,51,52,53] • Microinjection needle detection [39,48,53,54,55]	To avoid damaging the cell structure and improve the operational efficiency
	Rapid automatic focusing	• Image sharpness evaluation function [53,56] • Focus position search • Image sharpness global maximization search strategy [57,58,59]	
2. Recognize cell postures based on microscopic visuals and adjust the cell postures	Cell posture adjustment	Contact [42,60] and non-contact: micro-fluid, dielectric electrophoresis, magnetic field method, ultrasonic method	To resolve the problem in cell posture adjustment during the pre-puncturing stage, so that the injection needle can be kept away from the first polar body and guided to the ideal injection site
	Visual servoing control	• Position-based visual servoing control [60,61] • Image-based visual servoing control [39] • Trajectory planning [41]	
3. Perform rapid and effective puncture and quantitative injection of cells using a holding pipette and an injection needle-driving device	Actuator	• Piezoelectric ceramics [62,63,64] • Electrostrictive ceramics [65] • Other types of actuators are shown in Table 3	To ensure that the changes in the relationship between the applied force and chorion deformations caused by chorion softening during zebrafish embryo development do not affect the puncturing mechanism
	Sensor detection	• Micro-force sensor [38,66,67,68,69,70,71,72] • Micro-displacement sensor [73,74] • Visual sensor [75]	
	Cell models	• Young’s modulus [37,76] • Shear modulus [77,78] • Other cell models [64,79,80]	
	Puncturing mechanism	• Pulse puncturing [81] • Drilling movement [36,82] • Lateral vibration movement [83]	
	Microinjection	• Capillary pressure injection [1] • Pulse pressure injection [84,85] • Balanced pressure injection [45] • Capillary electrophoresis [86] • Capillary iontophoresis [87]	

**Table 2:** Methods for cell position and posture adjustment and their characteristics.

Method	Operational Principles		Advantages	Disadvantages	References
Mechanical contact method	Continuously hold and release the cell to adjust its position and posture		• Easy to operate • No additional equipment required	• Time consuming • Inefficient	[103]
	Use an injection needle to pluck the cells that are held in the holding tube		• Simple to operate • No additional equipment required	• Cell vulnerability	[104]
	Use a rotating device and a visual servoing system together to position the rotating cell at three points		• The ability to automatically adjust the cell position and posture	• Slightly lower operational efficiency	[60]
Non-contact method	Microfluidics	Fluid flow characteristics and interaction between forces	• Minor damage to cells • Rotation of cells to any position	• Complicated debugging • Low experimental efficiency	[42,105,106,107,108,109,110]
	Dielectrophoresis	Electric field force	• Fast operation • High positioning accuracy	• The influence of electric fields on the cells makes it difficult to set up a system	[111,112,113,114,115,116,117,118,119,120,121,122,123,124]
	Magnetic field	Magnetic force	• Easy to operate and control	• The influence of magnetic fields on the cells makes it difficult to set up a system.	[125,126,127,128,129]
	Ultrasonic	The action of acoustic radiation	• Easy to operate	• High local pressure and heat	[62,130,131]

**Table 3:** Comparison of the main actuator types.

Type	Operational Principles	Performance Features	Precision	References
Direct current motor	Electromagnetic effect	• Fast response but large force and displacement	Submicron	[137]
Piezoelectric ceramics	Piezoelectric effect	• Applicable in a wide range of frequencies but insensitive to temperature • No magnetic field influence but exhibits hysteresis	Sub-nanometer	[62,63,64,134,135]
Electrostrictive ceramic	Electrically induced effect	• Fast response but small force and displacement	Sub-nanometer	[65]
Shape memory alloy	Metal phase change	• Slow response and small force and displacement	Nano	[138]
Magnetostrictive material	Magnetic effect	• Good reliability, simple driving mode but exhibits hysteresis, low precision, poor response, and a tendency to overheat	Sub-nanometer	[139]
Giant magnetostrictive material	Magnetic effect	• Fast response but large force and displacement	Sub-nanometer	[140]
Ultrasonic motor	Piezoelectric effect Ultrasonic oscillation	• Fast response speed but large force and displacement	10 nm (linear) Seconds (rotary type)	[141]

**Table 4:** Measurement methods and characteristics of micro-force sensors.

Type	Detection Principle	Advantages	Disadvantages	Precision	References
Piezoelectric sensor	Piezoelectric effect of piezoelectric materials	• Wide band • High sensitivity • High signal-to-noise ratio • Simple structure	• Poor output direct current response • Unsuitable for static measurement	μN–sub μN	[142]
Piezoresistive sensor	The relationship between force and resistance	• Proven detection method and good frequency response	• Modest signal-to-noise ratio • Complex structure • Temperature-sensitive	mN–sub mN	[143,144]
Capacitive transducer	The relationship between force and capacitance change between plates	• Simple structure • Good stability • High sensitivity	• Highly nonlinear strain	μN–sub μN	[66,69]
Strain gauge	The relationship between the shape variable and stress	• Simple structure	• Modest detection accuracy	mN	[145]
Polyvinylidene fluoride force transducer	Piezoelectric effect	• High linearity • High signal-to-noise ratio • Suitable for dynamic force induction	• Cannot work under high temperature	sub μN	[146,147,148]
Polydimethylsiloxane (PDMS) patch force transducer	Deviate from PDMS posts	• Easy to fabricate and can be used to study different types of cell characteristics	• Difficult to prepare	nN	[68,149]
Cantilever-based force sensor	Beam deflection	• Easy to fabricate and allows vision-enabled measurement of structural deformation	• Low sensitivity and precision • Difficult to detect static forces	nN	[150,151]
Cantilever force transducer with an indentation probe	Beam deflection	• Simple structure • High resolution, can be used to study the force response of cells under large deformation	• Complex mechanical structure	nN–pN	[71,152,153,154]
Miniature camera-based force sensor	Change in diffraction efficiency	• High resonance frequency	• Difficult to fabricate • Complex optical setup	N/A	[38]
Magnetic effect-based sensor	The compressive magnetic effect of magnetic materials	• High measurement accuracy	• Prone to be affected by the surrounding magnetic field	nN	[155]
Vision-based sensor	The relationship between stress and image deviation	• Non-contact measurement	• Strict requirements for image processing precision	mN–μN	[156,157]

## References

[1] Kuncova J., Kallio P. (2004). Challenges in capillary pressure microinjection. Conf. Proc. IEEE Eng. Med. Biol. Soc..

[2] Iritani A. (1991). Micromanipulation of gametes for in vitro assisted fertilization. Mol. Reprod. Dev..

[3] Rols M.P. (2006). Electropermeabilization, a physical method for the delivery of therapeutic molecules into cells. Biochim. Biophys. Acta.

[4] Sakaki K., Dechev N., Burke R.D., Park E.J. (2009). Development of an autonomous biological cell manipulator with single-cell electroporation and visual servoing capabilities. IEEE Trans. Biomed. Eng..

[5] Walther W., Stein U. (2000). Viral Vectors for Gene Transfer. Drugs.

[6] Kalia Y.N., Naik A., Garrison J., Guy R.H. (2004). Iontophoretic drug delivery. Adv. Drug Deliv. Rev..

[7] Lin M.T.S., Pulkkinen L., Kyonggeun Y. (2002). The gene gun: Current application in cutaneous gene therapy. J. Pak. Assoc. Dermatol..

[8] Sundaram J., Mellein B.R., Mitragotri S. (2003). An experimental and theoretical analysis of ultrasound-induced permeabilization of cell membranes. Biophys. J..

[9] Unger E.C., Hersh E., Vannan M., McCreery T. (2001). Gene Delivery Using Ultrasound Contrast Agents. Echocardiography.

[10] Lavitrano M., Busnelli M., Cerrito M.G., Giovannoni R., Manzini S., Vargiolu A. (2006). Sperm-mediated gene transfer. Reprod. Fertil. Dev..

[11] Liu F., Song Y.K., Liu D. (1999). Hydrodynamics-based transfection in animals by systemic administration of plasmid DNA. Gene Ther..

[12] Zhang G., Budker V., Wolff J.A. (1999). High Levels of Foreign Gene Expression in Hepatocytes after Tail Vein Injections of Naked Plasmid DNA. Hum. Gene Ther..

[13] Ionescu-Zanetti C., Blatz A., Khine M. (2008). Electrophoresis-assisted single-cell electroporation for efficient intracellular delivery. Biomed. Microdevices.

[14] Olofsson J., Nolkrantz K., Ryttsén F., Lambie B.A., Weber S.G., Orwar O. (2010). Single-cell electroporation. Anal. Bioanal. Chem..

[15] Gao X., Kim K.-S., Liu D. (2007). Nonviral gene delivery: What we know and what is next. AAPS J..

[16] Luo D., Saltzman W.M. (2000). Synthetic DNA delivery systems. Nat. Biotechnol..

[17] Wu Y.C., Wu T.H., Clemens D.L., Lee B.Y., Wen X., Horwitz M.A., Teitell M.A., Chiou P.Y. (2015). Massively parallel delivery of large cargo into mammalian cells with light pulses. Nat. Methods.

[18] Wang Y., Yang Y., Yan L., Kwok S.Y., Li W., Wang Z., Zhu X., Zhu G., Zhang W., Chen X. (2014). Poking cells for efficient vector-free intracellular delivery. Nat. Commun..

[19] Navarro J., Risco R., Toschi M., Schattman G. (2008). Gene Therapy and Intracytoplasmatic Sperm Injection (ICSI)—A Review. Placenta.

[20] Graf S.F., Madigou T., Li R., Chesné C., Stemmer A., Knapp H.F. (2011). Fully Automated Microinjection System for Xenopus laevis Oocytes With Integrated Sorting and Collection. J. Lab. Autom..

[21] Villefranc J.A., Amigo J., Lawson N.D. (2007). Gateway compatible vectors for analysis of gene function in the zebrafish. Dev. Dyn..

[22] Van der Sar A.M., Musters R.J.P., van Eeden F.J.M., Appelmelk B.J., Vandenbroucke-Grauls C.M.J.E., Bitter W. (2003). Zebrafish embryos as a model host for the real time analysis of Salmonella typhimurium infections. Cell. Microbiol..

[23] Görge G., Nagel R. (1990). Toxicity of lindane, atrazine, and deltamethrin to early life stages of zebrafish (Brachydanio rerio). Ecotoxicol. Environ. Saf..

[24] Xu H., Yang M., Qiu W., Pan C., Wu M. (2013). The impact of endocrine-disrupting chemicals on oxidative stress and innate immune response in zebrafish embryos. Environ. Toxicol. Chem..

[25] Novoa B., Figueras A. (2012). Zebrafish: Model for the study of inflammation and the innate immune response to infectious diseases. Adv. Exp. Med. Biol..

[26] Xiang J., Yang H., Che C., Zou H., Yang H., Wei Y., Quan J., Zhang H., Yang Z., Lin S. (2009). Identifying tumor cell growth inhibitors by combinatorial chemistry and zebrafish assays. PLoS ONE.

[27] Sun Y., Nelson B.J. (2001). Microrobotic cell injection. Proc. IEEE Int. Conf. Robot. Autom..

[28] Matsuoka H., Komazaki T., Mukai Y., Shibusawa M., Akane H., Chaki A., Uetake N., Saito M. (2005). High throughput easy microinjection with a single-cell manipulation supporting robot. J. Biotechnol..

[29] Ammi M., Ferreira A. (2005). Realistic visual and haptic rendering for biological-cell injection. Proc. IEEE Int. Conf. Robot. Autom..

[30] Li X., Zong G., Bi S. Development of Global Vision System for Biological Automatic Micro-Manipulation System. Proceedings of the 2001 ICRA IEEE International Conference on Robotics and Automation (Cat. No.01CH37164).

[31] Kapoor A., Taylor R.H. Preliminary Experiments in RobotMuman Cooperative Microinjection. Proceedings of the 2003 IEEE/RSJ International Conference on Intelligent Robots and Systems (IROS 2003) (Cat. No.03CH37453).

[32] Huang H., Sun D., Mills J.K., Li W.J., Cheng S.H. (2009). Visual-Based Impedance Control of Out-of-Plane Cell Injection Systems. Science.

[33] Huang H., Sun D., Mills J.K., Li W.J. Visual-based impedance force control of three-dimensional cell injection system. Proceedings of the 2007 IEEE International Conference on Robotics and Automation.

[34] Xu Q. (2015). Design, Fabrication, and Testing of an MEMS Microgripper with Dual-Axis Force Sensor. IEEE Sens..

[35] Wang G., Xu Q. (2017). Design and Precision Position/Force Control of a Piezo-Driven Microinjection System. IEEE/ASME Trans. Mechatron..

[36] Huang H.B., Su H., Chen H.Y., Mills J.K. (2011). Piezoelectric driven non-toxic injector for automated cell manipulation. Stud. Health Technol. Inform..

[37] Kim D.-H., Sun Y., Yun S., Kim B., Hwang C.N., Lee S.H., Nelson B.J. (2004). Mechanical property characterization of the zebrafish embryo chorion. Conf. Proc. IEEE Eng. Med. Biol. Soc..

[38] Kim D.-H., Hwang C.N., Sun Y., Lee S.H., Kim B., Nelson B.J. (2006). Mechanical analysis of chorion softening in prehatching stages of zebrafish embryos. IEEE Trans. Nanobiosci..

[39] Liu X., Shi Q., Wang H., Sun T., Yu N., Huang Q., Fukuda T. (2018). Automated Fluidic Assembly of Microvessel-Like Structures Using a Multimicromanipulator System. IEEE/ASME Trans. Mechatronics.

[40] Wang W., Liu X., Gelinas D., Ciruna B., Sun Y. (2007). A fully automated robotic system for microinjection of zebrafish embryos. PLoS ONE.

[41] Nan Z., Xu Q. Multiple-cell recognition and path planning for robotic microinjection system. Proceedings of the 2017 36th Chinese Control Conference (CCC).

[42] Wang Z., Feng C., Muruganandam R., Mathew J., Wong P.C., Ang W.T., Tan S.Y.M., Latt W.T. A fully automated robotic system for three-dimensional cell rotation. Proceedings of the 2016 IEEE International Conference on Robotics and Automation (ICRA).

[43] Lu Z., Peter C.Y.C., Nam J.H., Ge R., Lin W. A micromanipulation system with dynamic force-feedback for automatic batch microinjection. 2013, 17, 14–15.

[44] Huang H., Sun D., Mills J.K., Cheng S.H. (2008). Integrated vision and force control in suspended cell injection system: Towards automatic batch biomanipulation. Proc. IEEE Int. Conf. Robot. Autom..

[45] Wang Y., Sun M., Zhao X., Zhao B. Autonomous operating process for zebrafish embryo injection. Proceedings of the 2012 International Conference on Manipulation, Manufacturing and Measurement on the Nanoscale (3M-NANO).

[46] Liu X., Lu Z., Sun Y. (2011). Orientation control of biological cells under inverted microscopy. IEEE/ASME Trans. Mechatron..

[47] Sun Y., Nelson B.J. (2002). Biological Cell Injection Using an Autonomous MicroRobotic System. Int. J. Robot. Res..

[48] Wang W.H., Liu X.Y., Sun Y. (2007). Contact detection in microrobotic manipulation. Int. J. Robot. Res..

[49] Sun M., Zhao X., Cheng X., Sun C., Lu G. Key technologies of micro-manipulation system oriented complex task. Proceedings of the CCC 2010 29th Chinese Control Conference.

[50] Huang H.B., Sun D., Mills J.K., Cheng S.H. (2009). Robotic cell injection system with position and force control: Toward automatic batch biomanipulation. IEEE Trans. Robot..

[51] Leavers V.F. (1993). Which Hough Transform?. CVGIP Image Underst..

[52] Zhang H., Liang C., Wang Y. Chord midpoint randomized Hough transform for the cell image segmentation. Proceedings of the 2011 Cross Strait Quad-Regional Radio Science and Wireless Technology Conference.

[53] Xie Y., Zeng F., Xi W., Zhou Y., Liu H., Chen M. (2016). A robot-assisted cell manipulation system with an adaptive visual servoing method. Micromachines.

[54] Mattos L., Grant E., Thresher R. (2006). Semi-automated blastocyst microinjection. Proc. IEEE Int. Conf. Robot. Autom..

[55] Yu J., Zhao Q., Cui M., Sun M., Zhao X. Robotic Donor Cell Injection in Somatic Cell Nuclear Transfer (SCNT). In Proceeding of the 11th World Congress on Intelligent Control and Automation.

[56] Sun Y., Duthaler S., Nelson B.J. (2004). Autofocusing in computer microscopy: Selecting the optimal focus algorithm. Microsc. Res. Technol..

[57] Nathaniel N.K.C., Neow P.A., Ang M.H. Practical issues in pixel-based autofocusing for machine vision. Proceedings of the 2001 ICRA. IEEE International Conference on Robotics and Automation (Cat. No.01CH37164).

[58] Sun M., Zong G., Yu Z., Bi S., Yu J. (2005). Automatic focusing system of micro-vision based on image analysis. J. Beijing Univ. Aeronaut. Astronaut..

[59] Ren S.-G., Li J.-W., Xie L.L. (2003). Automatic focusing technique based on gray scale difference method. Opto-Electron. Eng..

[60] Wang Z., Latt W.T., Tan S.Y.M., Ang W.T. (2015). Visual servoed three-dimensional cell rotation system. IEEE Trans. Biomed. Eng..

[61] Zhuang S., Lin W., Gao H., Shang X., Li L. (2017). Visual servoed zebrafish larva heart microinjection system. IEEE Trans. Ind. Electron..

[62] Feng L., Song B., Zhang D., Jiang Y., Arai F. (2018). On-chip Tunable Cell Rotation Using Acoustically Oscillating Asymmetrical Microstructures. Micromachines.

[63] Zhou M., Fan Z., Ma Z., Zhao H., Guo Y., Hong K., Li Y., Liu H., Wu D. (2017). Design and experimental research of a novel stick-slip type piezoelectric actuator. Micromachines.

[64] Qin X., Zhao X., Che X., Fang Y. Modeling of quantitative microinjection and adaptive control. Proceedings of the 30th Chinese Control Conference CCC 2011.

[65] Hom C.L., Shankar N. (1996). A finite element method for electrostrictive ceramic devices. Int. J. Solids Struct..

[66] Sun Y., Wan K.-T., Roberts K.P., Bischof J.C., Nelson B.J. (2003). Mechanical property characterization of mouse zona pellucida. IEEE Trans. Nanobiosci..

[67] Sun Y., Nelson B.J. (2007). MEMS capacitive force sensors for cellular and flight biomechanics. Biomed. Mater..

[68] Liu X., Sun Y., Wang W., Lansdorp B.M. (2007). Vision-based cellular force measurement using an elastic microfabricated device. J. Micromech. Microeng..

[69] Liu X., Kim K., Zhang Y., Sun Y. (2009). Nanonewton force sensing and control in microrobotic cell manipulation. Int. J. Robot. Res..

[70] Kim D.-H., Yun S., Kim B. Mechanical force response of single living cells using a microrobotic system. Proceedings of the IEEE International Conference on Robotics and Automation.

[71] Zhang X.J., Zappe S., Bernstein R.W., Sahin O., Chen C.C., Fish M., Scott M.P., Solgaard O. (2004). Micromachined silicon force sensor based on diffractive optical encoders for characterization of microinjection. Sens. Actuators A Phys..

[72] Muntwyler S., Beyeler F., Nelson B.J. (2010). Three-axis micro-force sensor with tunable force range and sub-micronewton measurement uncertainty. Proc. IEEE Int. Conf. Robot. Autom..

[73] Feng J.-Y., Ye X.-Y., Chen F., Shang Y.-F. (2012). Interferometric displacement measurement of microcantilevers based on integrated dual gratings. Guangxue Jingmi Gongcheng/Opt. Precis. Eng..

[74] Ergenc A.F., Olgac N. Micro-pipette Motion Detection by using Optical Fiber Sensors. Proceedings of the IEEE 31st Annual Northeast Bioengineering Conference.

[75] Karimirad F., Shirinzadeh B., Zhong Y., Smith J., Mozafari M.R. Modelling a Precision Loadcell using Neural Networks for Vision–Based Force Measurement in Cell Micromanipulation. Proceedings of the 2013 IEEE/ASME International Conference on Advanced Intelligent Mechatronics.

[76] Tan Y., Sun D., Huang W., Cheng S.H. (2008). Mechanical modeling of biological cells in microinjection. IEEE Trans. Nanobiosci..

[77] Sit P.S., Spector A.A., Lue A.J.C., Popel A.S., Brownell W.E. (1997). Micropipette aspiration on the outer hair cell lateral wall. Biophys. J..

[78] Spector A.A., Brownell W.E., Popel A.S. (1996). A model for cochlear outer hair cell deformations in micropipette aspiration experiments: An analytical solution. Ann. Biomed. Eng..

[79] Xie Y., Sun D., Liu C., Cheng S.H., Liu Y.H. A force control based cell injection approach in a bio-robotics system. Proceedings of the 2009 IEEE International Conference on Robotics and Automation.

[80] Zhang Y.L., Han M.L., Vidyalakshmi J., Shee C.Y., Ang W.T. (2009). Automatic control of mechanical forces acting on cell biomembranes using a vision-guided microrobotic system in computer microscopy. J. Microsc..

[81] Kimura Y., Yanagimachi R. (1995). Intracytoplasmic sperm injection in the mouse. Biol. Reprod..

[82] Johnson W., Dai C., Liu J., Wang X., Luu D.K., Zhang Z., Ru C., Zhou C., Tan M., Pu H. (2018). A Flexure-Guided Piezo Drill for Penetrating the Zona Pellucida of Mammalian Oocytes. IEEE Trans. Biomed. Eng..

[83] Huang H., Mills J.K., Sun D. (2011). A universal piezo-driven ultrasonic cell microinjection system. Biomed. Microdevices.

[84] Zhang W.Y., Hou L., Mu L., Zhu L. (2004). Femtoliter micro injector using digital microfluidic control. Conf. Microfluid. BioMEMS Med. Microsyst. II.

[85] Lee S., Jeong W., Beebe D.J. (2003). Microfluidic valve with cored glass microneedle for microinjection. Lab Chip.

[86] Kim J.A., Cho K., Shin M.S., Lee W.G., Jung N., Chung C., Chang J.K. (2008). A novel electroporation method using a capillary and wire-type electrode. Biosens. Bioelectron..

[87] Sharma S., Parvez N., Sharma P.K. (2015). Iontophoresis—Models and Applications: A Review. Afr. J. Basic Appl. Sci..

[88] Liu X., Fernandes R., Gertsenstein M., Perumalsamy A., Lai I., Chi M., Moley K.H., Greenblatt E., Jurisica I., Casper R.F. (2011). Automated microinjection of recombinant BCL-X into mouse zygotes enhances embryo development. PLoS ONE.

[89] He J., Ge H., Wang Y. (2009). Survey on the Methods of Image Segmentation Research. Comput. Eng. Sci..

[90] Canny J. (1986). A Computational Approach to Edge Detection. IEEE Trans. Pattern Anal. Mach. Intell..

[91] Duda R.O., Hart P.E. (1972). Use of the Hough transformation to detect lines and curves in pictures. Commun. ACM.

[92] Morales D.A., Bengoetxea E., Larrañaga P. (2008). Selection of human embryos for transfer by Bayesian classifiers. Comput. Biol. Med..

[93] Du Q., Zhang Q., Tian L., Wu Z. Object Detection and Tracking for a Vision Guided Automated Suspended Cell Injection Process. Proceedings of the 2010 IEEE International Conference on Mechatronics and Automation, Xi’an.

[94] Liu X., Fernandes R., Jurisicova A., Casper R.F., Sun Y. (2010). In situ mechanical characterization of mouse oocytes using a cell holding device. Lab Chip.

[95] Otsu N. (1979). A Threshold Selection Method from Gray-Level Histograms. IEEE Trans. Syst. Man. Cybern..

[96] Mattos L., Grant E., Thresher R. (2006). Speeding up video processing for blastocyst microinjection. IEEE Int. Conf. Intell. Robot. Syst..

[97] Wang W.H., Liu X.Y., Sun Y. (2009). High-throughput automated injection of individual biological cells. IEEE Trans. Autom. Sci. Eng..

[98] Wang W.H., Liu X.Y., Sun Y. Autonomous Zebrafish Embryo Injection Using a Microrobotic System. Proceedings of the 2007 IEEE International Conference on Automation Science and Engineering.

[99] Zong G.H., Sun M.L., Bi S.S., Dong D. (2006). Research on wavelet based autofocus evaluation in micro-vision. Chin. J. Aeronaut..

[100] Yu B., Yang Z., Tian F., Dong J., Jiang B. (2010). Definition Evaluation of Auto Focus in Micro-vision Based on the Macro-micro Dual-drive. Trans. Chin. Soc. Agric. Mach..

[101] Chen L.-G., Wang M.-Y., Yang Z.-L., Rong W.-B. (2010). Fast autofocus method for microscopic computer vision. Guangxue Jingmi Gongcheng/Opt. Precis. Eng..

[102] Zhou L.P., Sun Z.J., Zhang Q. (2013). Auto-focusing and control of micro-vision system. Opt. Precis. Eng..

[103] Zhang Y., Ballas C.B., Rao M.P. Towards ultrahigh throughput microinjection: MEMS-based massively-parallelized mechanoporation. Proceedings of the 2012 Annual International Conference of the IEEE Engineering in Medicine and Biology Society.

[104] Anis Y.H., Holl M.R., Meldrum D.R. (2010). Automated selection and placement of single cells using vision-based feedback control. IEEE Trans. Autom. Sci. Eng..

[105] Aoyama H., Chiba N., Fuchiwaki O., Misaki D., Usuda T. Non-contact Bio Cell Manioulation by Nonlinear Micro Flow Around the Vibrated Pipette on Micro Robot. Proceedings of the 21st Annual Meeting of the American Society for Precision Engineering, ASPE 2006.

[106] Erdil E., Topalli K., Esmaeilzad N.S., Zorlu Ö., Kulah H., Aydin Civi O. (2015). Reconfigurable nested ring-split ring transmitarray unit cell employing the element rotation method by microfluidics. IEEE Trans. Antennas Propag..

[107] Wang Z., Feng C., Muruganandam R., Ang W.T., Tan S.Y.M., Latt W.T. (2016). Three-dimensional cell rotation with fluidic flow-controlled cell manipulating device. IEEE/ASME Trans. Mechatron..

[108] Leung C., Lu Z., Zhang X.P., Sun Y. (2012). Three-dimensional rotation of mouse embryos. IEEE Trans. Biomed. Eng..

[109] Tang H., Li Y., Xiao X. A novel flexure-based dual-arm robotic system for high-throughput biomanipulations on micro-fluidic chip. Proceedings of the 2013 IEEE/RSJ International Conference on Intelligent Robots and Systems.

[110] Shin Y.K., Kim Y., Kim J. Automated microfluidic system for orientation control of mouse embryos. Proceedings of the 2013 IEEE/RSJ International Conference on Intelligent Robots and Systems.

[111] Huang L., Tu L., Zeng X., Mi L., Li X., Wang W. Towards on-chip single cell manipulation of trap and rotation. Proceedings of the 2016 International Conference on Manipulation, Automation and Robotics at Small Scales (MARSS).

[112] Zhou J.-H., Gong Z., Li Y.-M. (2007). Micromanipulation by Means of optical Tweezers and Dielectrophoresis Technologies. Acta Laser Biol. Sin..

[113] Ouyang M., Zhang G., Li W.J., Liu W.K. Self-induced rotation of pigmented cells by dielectrophoretic force field. Proceedings of the 2011 IEEE International Conference on Robotics and Biomimetics.

[114] Park J., Jung S.-H., Kim Y.-H., Kim B., Lee S.-K., Ju B., Lee K.-L. An integrated bio cell processor for single embryo cell manipulation. Proceedings of the 2004 IEEE/RSJ International Conference on Intelligent Robots and Systems (IROS) (IEEE Cat. No.04CH37566).

[115] Jen C.-P., Chen T.-W. (2009). Trapping of cells by insulator-based dielectrophoresis using open-top microstructures. Microsyst. Technol..

[116] Hunt T.P., Westervelt R.M. (2006). Dielectrophoresis tweezers for single cell manipulation. Biomed. Microdevices.

[117] Arai F., Kawaji A., Luangjarmekorn P., Fukuda T., Itoigawa K. Three-dimensional bio-micromanipulation under the microscope. Proceedings of the 2001 ICRA. IEEE International Conference on Robotics and Automation (Cat. No.01CH37164).

[118] Wang C.-C., Lan K.-C., Chen M.-K., Wang M.-H., Jang L.-S. (2013). Adjustable trapping position for single cells using voltage phase-controlled method. Biosens. Bioelectron..

[119] Jiang C., Mills J.K. Development of a cell orientation control system for mouse embryo using electro-rotation. Proceedings of the 2014 IEEE International Conference on Mechatronics and Automation.

[120] Holzapfel C., Vienken J., Zimmermann U. (1982). Rotation of cells in an alternating electric field theory and experimental proof. J. Membr. Biol..

[121] Benhal P., Chase J.G., Gaynor P., Oback B., Wang W. (2014). AC electric field induced dipole-based on-chip 3D cell rotation. Lab Chip.

[122] Jones T.B. (2003). Basic Theory of Dielectrophoresis and Electrorotation. IEEE Eng. Med. Biol. Mag..

[123] Huang L., Zhao P., Bian S., Shi G., Liu P., Zong S., Wang W. A novel BioMEMS device for efficient on-chip single cell loading and 3D rotation. Proceedings of the 2017 IEEE 30th International Conference on Micro Electro Mechanical Systems (MEMS).

[124] Huang L., Tu L., Zeng X., Mi L., Li X., Wang W. (2016). Study of a microfluidic chip integrating single cell trap and 3D stable rotation manipulation. Micromachines.

[125] De Vries A.H.B., Krenn B.E., Van Driel R., Kanger J.S. (2005). Micro magnetic tweezers for nanomanipulation inside live cells. Biophys. J..

[126] Feng L., Turan B., Ningga U., Arai F. Three dimensional rotation of bovine oocyte by using magnetically driven on-chip robot. Proceedings of the 2014 IEEE/RSJ International Conference on Intelligent Robots and Systems.

[127] Winkleman A., Gudiksen K.L., Ryan D., Whitesides G.M., Greenfield D., Prentiss M. (2004). A magnetic trap for living cells suspended in a paramagnetic buffer. Appl. Phys. Lett..

[128] Floyd S., Pawashe C., Sitti M. (2009). Two-dimensional contact and noncontact micromanipulation in liquid using an untethered mobile magnetic microrobot. IEEE Trans. Robot..

[129] Rodríguez-Villarreal A.I., Tarn M.D., Madden L.A., Lutz J.B., Greenman J., Samitier J., Pamme N. (2011). Flow focussing of particles and cells based on their intrinsic properties using a simple diamagnetic repulsion setup. Lab Chip.

[130] Oberti S., Neild A., Dual J. (2007). Manipulation of micrometer sized particles within a micromachined fluidic device to form two-dimensional patterns using ultrasound. J. Acoust. Soc. Am..

[131] Läubli N., Shamsudhin N., Ahmed D., Nelson B.J. (2017). Controlled Three-dimensional Rotation of Single Cells Using Acoustic Waves. Procedia CIRP.

[132] Kim D.-H., Haake A., Sun Y., Neild A.P., Ihm J.-E., Dual J., Hubbell J.A., Ju B.-K., Nelson B.J. (2004). High-throughput cell manipulation using ultrasound fields. Conf. Proc. IEEE Eng. Med. Biol. Soc..

[133] Becattini G., Mattos L.S., Caldwell D.G. (2014). A fully automated system for adherent cells microinjection. IEEE J. Biomed. Heal. Inf..

[134] Liu J., Siragam V., Gong Z., Chen J., Fridman M.D., Leung C., Lu Z., Ru C., Xie S., Luo J. (2015). Robotic adherent cell injection for characterizing cell-cell communication. IEEE Trans. Biomed. Eng..

[135] Xie Y., Sun D., Liu C., Cheng S.H. An adaptive impedance force control approach for robotic cell microinjection. Proceedings of the 2008 IEEE/RSJ International Conference on Intelligent Robots and Systems.

[136] Wang G., Xu Q. Position and force switching control of a piezo-driven microinjection system. Proceedings of the 2016 35th Chinese Control Conference (CCC).

[137] Tǎtar O., Mândru D., Ardelean I. (2007). Development of mobile minirobots for in pipe inspection tasks. Mechanika.

[138] Gong F.F., Shen H.M., Wang Y.N. (2013). Structures and defects induced during annealing of sputtered near-equiatomic NiTi shape memory thin films Structures and defects induced during annealing of sputtered near-equiatomic NiTi shape memory thin films. Appl. Phys. Lett..

[139] Ishihara H., Aral F., Fukuda T. (1996). Micro mechatronics and micro actuators. IEEE/ASME Trans. Mechatron..

[140] Clark A.E. (1980). Chapter 7 Magnetostrictive rare earth-Fe_2_ compounds. Handbook of Ferromagnetic Materials.

[141] Petit L., Lebrun L., Briot R., Gonnard P. Estimation of available performances of ultrasonic motors. Proceedings of the SPIE—The International Society for Optical Engineering.

[142] Kim D.H., Kim B., Kang H. (2004). Development of a piezoelectric polymer-based sensorized microgripper for microassembly and micromanipulation. Microsyst. Technol..

[143] Arai F., Kawaji A., Sugiyama T., Onomura Y., Ogawa M., Fukuda T., Iwata H., Itoigawa K. 3D micromanipulation system under microscope. Proceedings of the MHA’98 1998 International Symposium on Micromechatronics and Human Science.-Creation of New Industry-(Cat. No.98TH8388).

[144] Tan J.L., Tien J., Pirone D.M., Gray D.S., Bhadriraju K., Chen C.S. (2003). Cells lying on a bed of microneedles: An approach to isolate mechanical force. Proc. Natl. Acad. Sci. USA.

[145] Carrozza M.C., Eisinberg A., Menciassi A., Campolo D., Micera S., Dario P. (2000). Towards a force-controlled microgripper for assembling biomedical microdevices. J. Micromech. Microeng..

[146] Zhang R., Chu J., Wang H., Chen Z. (2013). A multipurpose electrothermal microgripper for biological micro-manipulation. Microsyst. Technol..

[147] Wei M., Gao Y., Li X., Serpe M.J. (2017). Stimuli-responsive polymers and their applications. Polym. Chem..

[148] Fung C.K.M., Elhajj I., Li W.J., Xi N. A 2-D PVDF force sensing system for micro-manipulation and micro-assembly. Proceedings of the 2002 IEEE International Conference on Robotics and Automation (Cat. No.02CH37292).

[149] Xie Y., Sun D., Tse H.Y.G., Liu C., Cheng S.H. (2011). Force sensing and manipulation strategy in robot-assisted microinjection on zebrafish embryos. IEEE/ASME Trans. Mechatron..

[150] Pelham R.J., Wang Y.-L. (1999). High Resolution Detection of Mechanical Forces Exerted by Locomoting Fibroblasts on the Substrate. Mol. Biol. Cell.

[151] Dembo M., Wang Y.L. (1999). Stresses at the cell-to-substrate interface during locomotion of fibroblasts. Biophys. J..

[152] Yang M.T., Sniadecki N.J., Chen C.S. (2007). Geometric considerations of micro- To nanoscale elastomeric post arrays to study cellular traction forces. Adv. Mater..

[153] Ghibaudo M., Di Meglio J.-M., Hersen P., Ladoux B. (2011). Mechanics of cell spreading within 3D-micropatterned environments. Lab Chip.

[154] Sniadecki N.J., Anguelouch A., Yang M.T., Lamb C.M., Liu Z., Kirschner S.B., Liu Y., Reich D.H., Chen C.S. (2007). Magnetic microposts as an approach to apply forces to living cells. Proc. Natl. Acad. Sci. USA.

[155] Kleinke D.K., Uras H.M. (1994). A magnetostrictive force sensor. Rev. Sci. Instrum..

[156] Greminger M.A., Nelson B.J. (2004). Vision-Based Force Measurement. IEEE Trans. Pattern Anal. Mach. Intell..

[157] Li F.-D., Xu D., Shi Y.-L., Zhang Z.-T. Development of Vision-Based Force Measurement. Proceedings of the 31st Chinese Control Conference.

[158] Xie Y., Sun D., Liu C. Penetration Force Measurement and Control in Robotic Cell Microinjection. Proceedings of the 2009 IEEE/RSJ International Conference on Intelligent Robots and Systems.

[159] Wang G., Xu Q. (2017). Design and development of a piezo-driven microinjection system with force feedback. Adv. Robot..

[160] Hiramoto Y. (1963). Mechanical properties of sea urchin eggs. I. Surface force and elastic modulus of the cell membrane. Exp. Cell Res..

[161] Nakamura S., Hiramoto Y. (1978). Mechanical properties of the cell surface in starfish eggs. Dev. Growth Differ..

[162] Ladjal H., Hanus J.-L., Ferreira A. Methodologies of dynamic cell injection techniques using FEM biomechanical modeling. Proceedings of the 2008 2nd IEEE RAS & EMBS International Conference on Biomedical Robotics and Biomechatronics.

[163] Ergenc A.F., Li M.W., Toner M., Biggers J.D., Lloyd K.C.K., Olgac N. (2008). Rotationally oscillating drill (Ros-Drill©) for mouse ICSI without using mercury. Mol. Reprod. Dev..

[164] Wang Y., Sun M., Feng X., Wang Y.N., Zhao B., Zhao X. (2013). Automatic Operating Process for Zebrafish Embryo Injection. Int. J. Intell. Mechatron. Robot..

[165] Zhang L., Zhang Y., Yang Y., Chen J. (2004). Injection Volume Control by Thermal Way in Transgenic DNA Micro-Injection System. Chin. J. Mech. Eng..

